# Is that a *pibu* or a *pibo*? Children with reading and language deficits show difficulties in learning and overnight consolidation of phonologically similar pseudowords

**DOI:** 10.1111/desc.13023

**Published:** 2020-08-07

**Authors:** Jeffrey G. Malins, Nicole Landi, Kayleigh Ryherd, Jan C. Frijters, James S. Magnuson, Jay G. Rueckl, Kenneth R. Pugh, Rose Sevcik, Robin Morris

**Affiliations:** ^1^ Department of Psychology Georgia State University Atlanta GA USA; ^2^ Haskins Laboratories New Haven CT USA; ^3^ Department of Psychological Sciences University of Connecticut Storrs CT USA; ^4^ Faculty of Social Sciences Department of Child and Youth Studies Brock University St. Catharines ON Canada; ^5^ Department of Linguistics Yale University New Haven CT USA; ^6^ Department of Diagnostic Radiology Yale University School of Medicine New Haven CT USA

**Keywords:** artificial lexicon, consolidation, developmental language disorder, phonological competition, reading disability, word learning

## Abstract

Word learning is critical for the development of reading and language comprehension skills. Although previous studies have indicated that word learning is compromised in children with reading disability (RD) or developmental language disorder (DLD), it is less clear how word learning difficulties manifest in children with comorbid RD and DLD. Furthermore, it is unclear whether word learning deficits in RD or DLD include difficulties with offline consolidation of newly learned words. In the current study, we employed an artificial lexicon learning paradigm with an overnight design to investigate how typically developing (TD) children (*N* = 25), children with only RD (*N* = 93), and children with both RD and DLD (*N* = 34) learned and remembered a set of phonologically similar pseudowords. Results showed that compared to TD children, children with RD exhibited: (i) slower growth in discrimination accuracy for cohort item pairs sharing an onset (e.g. *pibu*‐*pibo*), but not for rhyming item pairs (e.g. *pibu*‐*dibu*); and (ii) lower discrimination accuracy for both cohort and rhyme item pairs on Day 2, even when accounting for differences in Day 1 learning. Moreover, children with comorbid RD and DLD showed learning and retention deficits that extended to unrelated item pairs that were phonologically dissimilar (e.g. *pibu*‐*tupa*), suggestive of broader impairments compared to children with only RD. These findings provide insights into the specific learning deficits underlying RD and DLD and motivate future research concerning how children use phonological similarity to guide the organization of new word knowledge.

## Graphical abstract

We employed an artificial lexicon learning paradigm with an overnight design to investigate how typically developing (TD) children, children with reading disability (RD), and children with both RD and developmental language disorder (DLD) learned and remembered a set of phonologically similar pseudowords. Compared to TD children, children with RD experienced difficulty learning and remembering phonologically similar items, whereas children with both RD and DLD experienced difficulty learning and remembering phonologically dissimilar items in addition to phonologically similar items. These findings suggest one of the specific deficits underlying RD and DLD is difficulty establishing robust phonological representations for newly learned words.

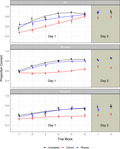

**Research Highlights**

- Compared to typically developing children, children with reading disability experienced difficulty discriminating between phonologically similar items when learning an artificial lexicon of spoken pseudowords
- During learning, children with comorbid reading disability and developmental language disorder experienced difficulty discriminating between phonologically dissimilar pseudowords in addition to phonologically similar pseudowords
- Analogous patterns of differences across learner groups were apparent when evaluating retention of spoken pseudowords following a period of offline consolidation
- These findings suggest one of the specific deficits underlying reading disability and developmental language disorder is difficulty establishing robust phonological representations for newly learned words

## 1 INTRODUCTION

The ability to learn new words is critical for academic success, as word knowledge lays a foundation for the development of reading and language comprehension skills (Perfetti, 2007). The process of word learning includes online binding of word‐level (lexical) information as well as consolidation into long‐term lexical memory (e.g. Davis & Gaskell, 2009). In children and young adults, learning of words, as well as offline consolidation, have been associated with written and oral language skills (James, Gaskell, & Henderson, 2019; Landi et al., 2018). Furthermore, word learning difficulties have been observed in children with either reading disability (RD) (Alt, Gray, Hogan, Schlesinger, & Cowan, 2019; Alt et al., 2017; Kimppa, Shtyrov, Partanen, & Kujala, 2018) or developmental language disorder (DLD)^1^ (Alt et al., 2019; Kan & Windsor, 2010), conditions that are characterized by impairments in reading (RD) or oral language skills (DLD) in the context of otherwise typical development. Yet, although RD and DLD are frequently comorbid (Pennington & Bishop, 2009), few studies have characterized how word learning deficits manifest in children with both RD and DLD (Alt et al., 2019). A better understanding of this relationship is important, as RD and DLD are at least partially etiologically distinct (Bishop & Snowling, 2004; Catts, Adlof, Hogan, & Weismer, 2005; Pennington & Bishop, 2009; Ramus, Marshall, Rosen, & van der Lely, 2013), and their comorbid occurrence may confer elevated risk for word learning difficulties (Alt et al., 2019).

Whereas RD is thought to arise primarily from deficits in phonological processing skills (Stanovich & Siegel, 1994), DLD is thought to stem from impairments in non‐phonological skills in addition to phonological processing deficits (Bishop & Snowling, 2004; Ramus et al., 2013). Therefore, although both RD and DLD may impact phonological processing, the potentially distinct sources of impairment that underlie RD and DLD may differentially influence how listeners resolve competition between phonologically similar words (Li et al., 2019; Magnuson et al., 2011). In the current study, we examined how typically developing (TD) children, children with RD, and children with both RD and DLD learned and remembered words that were phonologically related to one another in different ways. Critically, we employed a spoken artificial lexicon paradigm which allowed us to control for familiarity and exposure to individual items (Magnuson, Tanenhaus, Aslin, & Dahan, 2003). By using a variant of this paradigm that allowed us to measure word learning accuracy over consecutive days – that is, both during learning as well as after a period of offline consolidation – we were able to characterize groupwise differences that provide insights into the specific learning deficits underlying RD and DLD.

### 1.1 Word learning

Given the importance of word knowledge for academic success and the well‐known “rich get richer” impact of existing word knowledge on word learning (e.g. Cain & Oakhill, 2011; Stanovich, 1993), it is important that we gain a better understanding of the processes that limit word learning in children with RD and DLD. Word learning involves successful binding of phonological, semantic, and orthographic (for print) features associated with a word. According to the lexical quality hypothesis, weaknesses in the representation of (or binding among) any of these constituents can result in poor lexical knowledge, with potential secondary effects for more complex aspects of language processing (Perfetti, 2007; Perfetti, Landi, & Oakhill, 2005); however, the phonological level is the most widely studied in relation to both RD and DLD (Bishop & Snowling, 2004; Joanisse & Seidenberg, 1998; Liberman & Shankweiler, 1985; Stanovich & Siegel, 1994). To identify which aspects of word learning are atypical in children with reading and language difficulties, a number of studies have used novel word learning experiments, typically using one or more variants of a paired associate learning (PAL) paradigm. These approaches usually require participants to learn novel mappings between a phonological form and a visual symbol or object, thus loosely mimicking the configurational stage of word learning (mapping of a word label with an object referent). This approach affords an exploration of the processes involved during word learning while limiting the confound of existing word knowledge.

For children with RD, studies have found that PAL is most impaired (though not exclusively so) when the task demands tax phonology (Mayringer & Wimmer, 2000; Messbauer & de Jong, 2003). A recent example is Alt et al. (2017), who observed that children with RD were comparable to TD children on the PAL aspects of a word learning task, but struggled when asked to assess the phonology of a newly learned word. Furthermore, Litt, Wang, Sailah, Badcock, and Castles (2019) found that children with RD were impaired on visual‐to‐verbal learning (requiring input and output phonology) but not verbal‐to‐visual learning (requiring only input phonology); see also Litt and Nation (2014) for similar findings. This pattern of findings suggests that children with RD may only be impaired in PAL for tasks that have a phonological output demand.

In contrast, children with DLD appear to have broader difficulties with word learning, including visual (Alt, 2013; Collisson, Grela, Spaulding, Rueckl, & Magnuson, 2015), semantic (Alt & Plante, 2006; Gray, 2005), and phonological impairments (Dollaghan, 1987; Rice, Buhr, & Nemeth, 1990). These findings have contributed to theories that propose domain‐general learning and/or generalization deficits in children with DLD, including procedural learning deficits (Tomblin, Mainela‐Arnold, & Zhang, 2007; Ullman & Pierpont, 2005) and statistical learning deficits (Lammertink, Boersma, Wijnen, & Rispens, 2017). Notably, procedural and/or statistical learning deficits also have been observed in some studies of children and young adults with RD (Gabay, Thiessen, & Holt, 2015; Hung et al., 2018; Lum, Ullman, & Conti‐Ramsden, 2013; Nicolson & Fawcett, 1990). Taken together, these findings suggest that word learning deficits may be common to both DLD and RD, but that somewhat different mechanisms may underlie these deficits.

However, this interpretation is limited by the possibility that extant studies of word learning in RD or DLD may have unknowingly included children with both disorders, thus muddying conclusions about the relations between learning and either disorder (Adlof & Hogan, 2018). As argued by Adlof and Hogan (2018), explicit comparison of children with either or both of these two disorders in the same study is important for elucidating the similarities and differences in word learning profiles for children with language and reading problems. To date, only one study of word learning has compared children with RD with children with comorbid RD and DLD (Alt et al., 2019). In this study, across a number of word learning tasks, children with RD showed deficits in phonological tasks compared to TD children, whereas children with RD and DLD showed difficulties in both phonological and semantic tasks (Alt et al., 2019). The current study builds on this recent work by examining verbal‐visual paired associate learning in children with typical development, children with RD, and children with comorbid RD and DLD. Following the framework of Alt et al. (2019), the goal of this approach was to identify verbal‐visual PAL deficits associated with RD *and* any additional deficits that may be present in children with RD who also have comorbid language problems. In this way, we can begin to tease apart the distinctive RD‐associated word learning deficits from those that arise in the context of broader language impairments and resulting RD.

### 1.2 Consolidation

Several lines of research have found that memory for recently learned information is enhanced following a period of offline sleep or rest (e.g. Diekelmann & Born, 2010; Dumay, 2016). This is often termed *sleep‐associated* or *offline consolidation* in reference to the cortical consolidation that is presumed to undergird these effects (e.g. McClelland, McNaughton, & O’Reilly, 1995). With respect to word learning specifically, studies have shown that a period of sleep facilitates ‘lexicalization’, as demonstrated by enhanced lexical competition effects following sleep in both children and adults (e.g. Brown, Weighall, Henderson, & Gaskell, 2012; Davis, Di Betta, Macdonald, & Gaskell, 2009; Davis & Gaskell, 2009; Gaskell & Dumay, 2003; Wang et al., 2017).

Critically, several studies have shown that individuals with reading or language deficits show reduced benefits from offline consolidation of newly learned items. For example, McGregor et al. (2013) tested adults with and without DLD 12 hr, 24 hr, and 1 week after word learning, and found that the performance gap between TD and DLD widened as the time elapsed following training (and the number of nights of sleep) increased. More recently, Smith et al. (2018) measured overnight improvement in recall performance for newly learned words in TD children and children with RD. They observed that both groups of children showed overnight improvement in recall – although children with RD showed poorer retention overall – yet only the TD group showed correlations between sleep measures and recall performance. Furthermore, when the group of children with RD was compared with a control group of children who were matched in initial (Day 1) recall performance, the control group showed a boost in recall performance 1 week after training that was not observed in the group of children with RD.

Other studies have shown that the benefits of offline consolidation on word learning also may be positively correlated with individual differences in vocabulary knowledge among TD individuals. Indeed, Henderson, Devine, Weighall, and Gaskell (2015) observed increased word form recall following sleep for those with larger vocabulary (even after controlling for initial performance) and Landi et al. (2018) showed increased cortical consolidation effects (measured with fMRI) for individuals with larger vocabulary and better decoding skill. Interestingly, some research suggests that these language‐skill associated individual differences in offline consolidation may extend to language learning more generally. For example, Earle, Landi, and Myers (2018) observed reduced consolidation effects (measured using event related potentials, or ERPs) for adults with DLD (relative to TD) in a non‐native phoneme contrast learning paradigm. These findings motivate the current investigation of word learning in RD and RD with comorbid DLD in the context of an overnight design. In this way we can more fully specify the scope of word learning and/or consolidation deficits in children with RD and those with RD and DLD.

### 1.3 Phonological competition effects and artificial lexicon learning

Word learning and processing are intertwined. That is, the quality of word knowledge that is accumulated during learning influences how words are processed during subsequent encounters (Nation, 2014). Therefore, in the case of spoken word learning, we can gain insights into the quality of phonological representations in children with RD or DLD by evaluating the dynamics of spoken word processing (that is, the time course of lexical activation and phonological competition as words are learned).

The dynamics of spoken word processing are often evaluated using online methods such as eyetracking (e.g. Allopenna, Magnuson, & Tanenhaus, 1998) or ERPs that can be used to compare the time course of processing between words sharing different types of phonological relationships, such as pairs of items overlapping in word onset (cohort pairs) or word‐final information (rhyming pairs). For example, using eyetracking and the visual world paradigm (Tanenhaus, Spivey‐Knowlton, Eberhard, & Sedivy, 1995), Desroches, Joanisse, and Robertson (2006) observed how 8‐ to 10‐year‐old children processed words sharing onsets (cohort competitors; e.g. *candle*‐*candy*) or phonological rimes (e.g. *candle*‐*sandal*). When presented with pictures of items in an array, and asked to look at the picture that matched a spoken word, children with RD showed similar onset competition effects as TD children (both groups took longer to settle on target pictures when a picture of a cohort competitor was present compared to a condition in which all pictures were phonologically unrelated). However, whereas TD children also showed significant rhyme competition, children with RD did not. This was interpreted as a lack of sensitivity to rhyme similarity in children with RD during spoken word processing.

Using ERPs, Desroches, Newman, Robertson, and Joanisse (2013) followed up on their initial study by presenting 8‐ to 11‐year‐old children with pictures of items and asking whether subsequently presented spoken words matched or mismatched each picture. The authors observed that children with RD showed more exaggerated cohort mismatch effects compared to TD children during a later time window of the N400 component, which was attributed to greater difficulty overcoming competition between items sharing onset similarity. Furthermore, similar to Desroches et al. (2006), results revealed a groupwise difference in rhyme processing: TD children showed a characteristic reduction of a later portion of the N400 component for rhyming words (e.g. *cone*‐*bone*) compared to unrelated words (e.g. *cone*‐*fox*), yet children with RD did not show this effect (Desroches et al., 2013).

In DLD however, previous results have been considerably more mixed than in RD. Using eyetracking and the visual world paradigm, McMurray et al. (2010) observed that compared to TD individuals, adolescents with DLD (mean age of 17 years) showed increased looks to both cohort and rhyme competitors, but only during later stages of the time course of processing. Yet, using ERPs, Malins et al. (2013) observed a lack of an N400 rhyme effect in English‐speaking children with DLD (aged 8–12 years), suggesting that children with DLD did not treat rhyming words any differently than unrelated words. This finding differs still from Kornilov, Magnuson, Rakhlin, Landi, and Grigorenko (2015), who used ERPs to investigate spoken word recognition in Russian‐speaking children with and without DLD (aged 7–15 years) and observed groupwise differences for cohort and unrelated word pairs, but not pairs of words sharing word‐final phonological overlap (note that not all items with word‐final overlap were rhyming words, based on constraints in Russian).

These mixed results across studies could have arisen for multiple reasons, including differences in experimental methodologies, participant age, methods of defining DLD, and especially the potential presence of unmeasured RD within these DLD samples. Additionally, these previous studies examined words that were already familiar to the participants. Therefore, individual differences in familiarity and exposure to the different words may have affected the quality of phonological representations and ensuing competition effects. One way to control for these differences is to use a spoken artificial lexicon of pseudowords that balances for attendant psycholinguistic factors because all individuals are equally unfamiliar with the pseudowords at the onset of the study. By including items that share different phonological relationships and measuring competition among the different items in the set, the spoken artificial lexicon paradigm can capture the dynamics of word learning by indexing how the quality of phonological representations changes over time (Magnuson et al., 2003).

Using a closed set of pseudowords containing some items overlapping in either onset or rhyme (e.g. *pibu*‐*pibo*; *pibu*‐*dibu*), Magnuson et al. (2011) evaluated word learning in a group of university students as well as a community sample of young adults that tended to have lower than average reading scores (and may have had lower than average language scores as well). Compared to the university sample, the community sample showed an overall shallower increase in accuracy across trial blocks. In addition, eye movement patterns showed clear rhyme competition effects for the university sample, but no such rhyme competition in the community sample, suggesting reduced sensitivity to rhyme in those with poor reading. This finding complemented the patterns observed in children with RD in Desroches et al. (2006) and extended this previous work by offering evidence for phonological instability during the course of spoken word learning in young adults. This in turn motivated the current extension of this paradigm to evaluate the dynamics of word learning in children with RD and comorbid RD and DLD, as it provides a sensitive measure of input phonology that may reveal group differences in processing for specific aspects of sublexical phonology (i.e. onsets and rhymes).

### 1.4 The current study

In the current study, we evaluated learning and consolidation of an artificial lexicon in TD children, children with RD, and children with comorbid RD and DLD. This artificial lexicon was identical to the one used with adults in Magnuson et al. (2011), and consisted of a closed set of pseudowords that shared either word‐initial phonemes (cohort item pairs), word‐final phonemes (rhyme item pairs), or were phonologically unrelated. Our rationale for using this paradigm was that weaknesses in phonological representations may lead to differential patterns of interference for cohort and rhyme item pairs during spoken word recognition and potentially to differences in learning trajectories for these two stimulus types between learner groups. Specifically, consistent with previous literature (e.g. Desroches et al., 2013), we predicted that children with RD may show enhanced cohort competition (e.g. for item pairs such as *candy*‐*candle*; here *pibu*‐*pibo*) during initial processing, suggesting that although they are sensitive to onset similarity, pairs of items with overlapping onsets are more confusable. Over the course of learning, this may lead to reduced growth in discrimination accuracy for these items. For rhyme item pairs (*candle*‐*sandal*; here *pibu*‐*dibu*) on the other hand, greater interference has been observed for TD individuals, suggesting reduced sensitivity to this information in those with RD (e.g., Desroches et al., 2006; Magnuson et al., 2011). Therefore, over learning, this reduced sensitivity in RD could actually result in enhanced growth in discrimination accuracy during learning for rhyme item pairs relative to the TD group.

Learning data were analysed using growth curve models (Mirman, Dixon, & Magnuson, 2008) that included a comparison to unrelated item pairs as a baseline for learning. In these models, the predicted pattern of performance was reduced growth for cohort item pairs relative to unrelated item pairs for children with RD relative to TD children, and enhanced (or equivalent) growth for rhyme item pairs relative to unrelated item pairs for children with RD relative to TD children. For the comorbid RD and DLD group, our predictions were somewhat less specific; however, based on extant work (e.g. Alt et al., 2019) we predicted that this group would show broader impairments in learning across all three stimulus type conditions compared to children with only RD, who were predicted to show differences in learning trajectories compared to TD children for phonologically similar item pairs (i.e. cohort and rhyme item pairs) but not for phonologically dissimilar item pairs (i.e. unrelated item pairs). Finally, based on documented associations between reading and language skills and consolidation effects (e.g. Landi et al., 2018), we hypothesized that after a period of offline consolidation, analogous patterns of differences across learner groups also would be apparent. That is, we predicted that relative to TD children, children with RD would show poorer retention of cohort and rhyme item pairs but not unrelated item pairs (as measured by discrimination accuracy on the second day of testing), whereas children with comorbid RD and DLD would show poorer retention for all three stimulus types.

## 2 METHODS

### 2.1 Participants

Children participated in this experiment as part of a larger study concerning response to intervention for RD (Arrington et al., 2019; Malins et al., 2018). This study was approved by the Georgia State University/Georgia Tech Institutional Review Board, and all parents/students provided informed consent/assent prior to participation in the study. All data presented in the current report were collected prior to the onset of intervention. From a total sample of 167 children in 3rd or 4th grade, data from 15 participants was excluded for the following reasons: missing assessment scores required for designating RD, DLD, or ADHD status (described below) (three participants); mixed assessment scores such that the child did not meet criteria for inclusion in any group (six participants); did not complete all eight of the required blocks of trials of the artificial lexicon learning experiment (six participants). Thus, the final sample included 152 children (66 female, mean age of 9.28, *SD* age of 0.66; age range between 7.8 and 11.3 years).

The presence of DLD was established using the conceptual framework set out in (Tomblin, Records, & Zhang, 1996). If a participant was below a standard score of 85 on the Peabody Picture Vocabulary Test (PPVT‐4; Dunn & Dunn, 2007), the Test of Narrative Language Ability Index (TNL; Gillam & Pearson, 2004), or the Clinical Evaluation of Language Fundamentals Core Language Composite (CELF‐4; Semel, Wiig, & Secord, 2003), this was taken as evidence of difficulty acquiring developmentally appropriate language skills. If a particular child was below the critical standard score on two of these three measures, DLD status was assigned.

The presence of RD was established along similar lines. If a participant performed below a standard score of 85 on the Broad Skills or Basic Skills Clusters from the Woodcock‐Johnson III Tests of Achievement (WJ‐III; Woodcock, McGrew, & Mather, 2001), the Standardized Reading Inventory Reading Quotient (SRI‐2; Newcomer, 1999), or the Test of Word Reading Efficiency Composite Scale Score (TOWRE‐2; Torgesen, Wagner, & Rashotte, 2012), this was taken as evidence of having difficulty acquiring developmentally appropriate reading skills. Because each of these composites were based on multiple reading assessments, participants were classified as having RD if they were below the critical standard score on any one composite score. Participants in the TD group were at or above standard scores of 85 on all of the reading and language measures that were used for group classification. Descriptive statistics concerning these three groups of children are reported in Table 1.

**Table 1:** Characteristics of the three learner groups

	TD (*N* = 25; 11 F)		RD‐only (*N* = 93; 36 F)			RD + DLD (*N* = 34; 19 F)		
	Mean	*SD*	Mean	*SD*	*N* < 85^a^	Mean	*SD*	*N* < 85
Age	9.2	0.6	9.2	0.6		9.6	0.7	
WJ‐III Broad Reading	113.4	6.0	83.7	8.9	43	77.2	11.5	26
WJ‐III Basic Reading	113.3	6.4	88.2	7.3	26	82.8	9.1	18
SRI‐2	111.6	12.8	79.5	10.2	62	72.0	10.6	31
TOWRE‐2	107.0	9.5	73.9	8.3	88	70.3	8.8	32
PPVT‐4	117.4	11.6	100.2	12.4	5	79.0	7.8	29
TNL	114.8	10.3	96.6	10.6	8	80.0	8.8	24
CELF‐4 Core Language	116.1	8.4	91.6	11.8	21	73.7	9.0	30
WASI‐II FSIQ‐2	112.8	10.6	96.7	9.8		85.5	6.9	
% with ADHD diagnosis^b^	0%		38.7%			29.4%		

Abbreviations: CELF‐4: Clinical Evaluation of Language Fundamentals; PPVT‐4: Peabody Picture Vocabulary Test; SRI‐2: Standardized Reading Inventory; TNL: Test of Narrative Language; TOWRE‐2: Test of Word Reading Efficiency; WASI‐II FSIQ‐2: Wechsler Abbreviated Scale of Intelligence Full Scale IQ‐2; WJ‐III: Woodcock‐Johnson III Tests of Achievement.

^a^ The number of participants within the group that had an assessment score less than 85 (the critical score that was used for group classification).

^b^ As described in the text, ADHD diagnosis was determined using parent and teacher reports from the DBRS and SWAN; the percentage reported here concerns the number of children who met criterion for subtypes involving inattention.

Although not of primary interest in this study, we also included ADHD diagnosis as an additional variable in our post‐hoc analyses. This diagnosis was designated using criteria from the DSM‐V, and either the Disruptive Behavior Rating Scale (DBRS; Barkley & Murphy, 1998) or Strengths and Weaknesses of ADHD‐symptoms and Normal‐behaviour (SWAN; Swanson et al., 2012) parent and teacher ratings as the instrument. A child was classified as meeting criteria for a particular ADHD subtype (on either the DBRS or SWAN instruments) if rated by either their parent or teacher as having six or more of the core symptoms at a severe level, and if the other rater indicated a minimum three or more of the core symptoms at a moderate level. In Table 1 below, the percent meeting criteria for any subtypes that involved inattention is reported (e.g. combined presentation and predominantly inattentive presentation).

### 2.2 Stimuli

This spoken artificial lexicon learning task was identical to the one described in Magnuson et al. (2011). In this task, participants learned mappings between spoken labels and pictures of animals. The animals were all unusual and unlikely to be recognized by American children. The eight spoken labels were a closed set of two‐syllable pseudowords with a CVCV structure. This set consisted of the following items: /pibo/, /pibu/, /dibo/, /dibu/, /tupa/, /tupi/, /bupa/, and /bupi/. Each label was randomly mapped to a single animal for each participant. Cohort item pairs shared the same onset (e.g. *pibu*‐*pibo*); rhyme item pairs shared the same word‐final phonemes, but differed in onset consonant (e.g. *tupi*‐*bupi*); unrelated item pairs were either near neighbors (*dibo*‐*pibu*) or did not share any phonemes in common (e.g. *dibo*‐*tupa*).^2^

### 2.3 Procedure

Testing took place over the course of two school days (i.e. between the hours of 8 a.m. and 5 p.m.). The 2 days were consecutive for all participants except for six, for whom testing sessions were either 2 days (*N* = 4) or 3 days apart (*N* = 2) due to testing administration issues. On Day 1, participants completed six blocks of trials, each 24 trials in length, which were used to measure learning. On Day 2, participants completed two blocks of trials, each 24 trials in length, which were used to measure retention.

**Figure 1:**
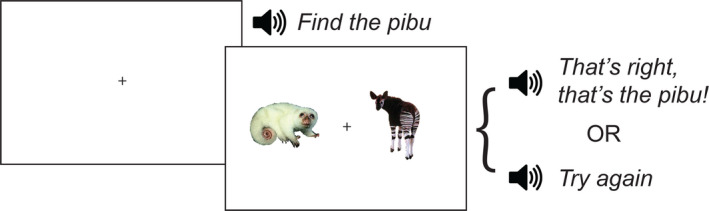
A sample trial from the artificial lexicon learning experiment

All trials had the same structure, as shown in Figure 1. First, a fixation cross appeared until the participant clicked on the cross. Then, two animal pictures appeared on the screen, and the participant simultaneously heard the instruction ‘Find the [*label*]’. Following this instruction, the participant clicked on one of the two animals. When they clicked on the correct animal, they heard positive feedback in the form of ‘That's right! That's the [*label*]’. When the participant clicked on the incorrect animal, they heard ‘Try again!’ and were allowed to click again until they selected the correct animal. Within each block of 24 trials, each of the eight items appeared as the target three times along with either its cohort or rhyme competitor or an unrelated item. Thus, each block had eight cohort, eight rhyme, and eight unrelated trials.

### 2.4 Analysis of learning data

Learning data from Day 1 were analysed using second order growth curve models (Mirman, 2014). The dependent measure was mean accuracy across the eight trials of each stimulus type within each block, and fixed effects included orthogonal polynomials for time (as defined by the sequential learning blocks, with intercept, linear, and quadratic terms all centred with respect to the time course of the six learning blocks), learner group (TD, RD‐only, RD + DLD), and stimulus type (cohort, rhyme, unrelated).

Random effects included random slopes and intercepts for participants, and random intercepts and slopes for stimulus types nested within participants; an initial model also included quadratic random‐effects terms for participants and stimulus types nested within participants, but these terms were removed because this initial model gave rise to a singular fit (Barr, Levy, Scheepers, & Tily, 2013).

In these models, fixed effects for the RD‐only and RD + DLD groups were estimated relative to the TD group, and fixed effects for cohort and rhyme item pairs were estimated relative to unrelated item pairs. Differences in growth curve parameter estimates were interpreted using the following guide: the (centred) intercept reflects the average amplitude across the entire time course (i.e. collapsed across time); the slope reflects linear growth across the time course, with larger values indicating steeper growth; the quadratic term indexes symmetric rise and fall about a central inflection point, with larger values indicating a sharper peak (Mirman, Dixon, & Magnuson, 2008). Negative slope values indicate a linear decrease over time, whereas negative quadratic terms indicate a parabolic curve that starts low, ascends to a central peak, and then falls. Therefore, lower intercepts, shallower slopes, and/or less negative quadratic terms were taken as indices of reduced growth, whereas greater intercepts, steeper slopes, and/or more negative quadratic terms were taken as indices of enhanced growth.

Growth curve analyses were carried out using version 1.1‐12 of the *lme4* package (Bates, Maechler, Bolker, & Walker, 2015) in R version 3.6.1. *p*‐values were computed using Satterthwaite's approximation for degrees of freedom method employed in *lmerTest* version 3.1‐0 (Kuznetsova, Brockhoff, & Christensen, 2017). For all models, optimization was performed using Bound Optimization by Quadratic Approximation (BOBYQA; Powell, 2009).

### 2.5 Analysis of retention data

Following analysis of the Day 1 learning data, we evaluated the extent to which learner group was associated with retention of items on Day 2. To do this, we extracted random effects estimates from the Day 1 learning growth curve models (intercept and slope terms). We then entered these into separate multiple regression models predicting Day 2 Block 1 accuracy for each of the three stimulus types. In this way, we assessed the extent to which the learner groups were predictive of retention even when accounting for differences in growth curve parameter estimates characterizing Day 1 learning. In these multiple regression models, we also included the amount of time elapsed between the Day 1 and Day 2 testing sessions as an additional predictor of non‐interest.

### 2.6 Data sharing

Preprocessed data and analysis scripts from this study are available on the following Open Science Framework project page: https://osf.io/az3tf/.

## 3 RESULTS

### 3.1 Learning of items on Day 1

**Figure 2:**
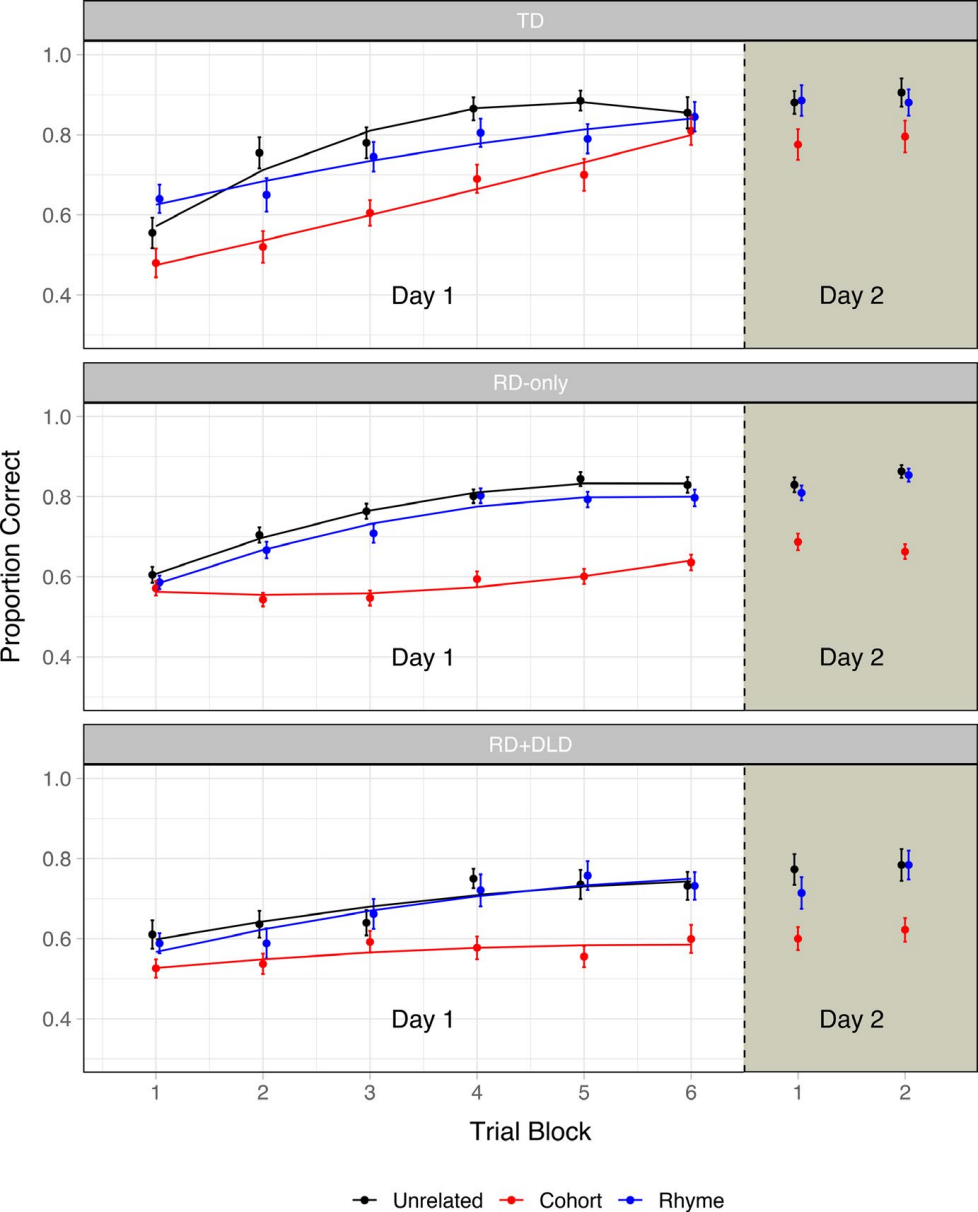
Accuracy across blocks for the artificial lexicon learning experiment. The first six blocks constitute the first day of learning, whereas the last two blocks (i.e. to the right of the dashed line) constitute the second day. Points and standard error bars represent raw data, whereas solid lines represent model fits

In Figure 2, growth curves for Day 1 learning of the spoken artificial lexicon are shown for each of the three learner groups, broken out by stimulus type. Parameter estimates for the growth curve models are reported in Table 2.

**Table 2:** Parameter estimates for the effect of learner group and stimulus type on Day 1 learning

	Estimate	*SE*	*df*	*t*	*p*
Intercept	0.783	0.023	291.8	33.35	<.0001
Linear	0.236	0.036	428.3	6.54	<.0001
Quadratic	−0.128	0.030	1,824.0	−4.29	<.0001
RD‐only: Intercept	−0.025	0.026	291.8	−0.93	.352
RD + DLD: Intercept	−0.099	0.031	291.8	−3.19	.002
Cohort: Intercept	−0.148	0.023	304.0	−6.52	<.0001
Rhyme: Intercept	−0.037	0.023	304.0	−1.61	.108
RD‐only: Linear	−0.047	0.041	428.3	−1.16	.245
RD + DLD: Linear	−0.115	0.048	428.3	−2.42	.016
RD‐only: Quadratic	0.058	0.034	1,824.0	1.74	.082
RD + DLD: Quadratic	0.104	0.039	1,824.0	2.64	.008
Cohort: Linear	0.036	0.046	304.0	0.78	.438
Rhyme: Linear	−0.056	0.046	304.0	−1.22	.225
Cohort: Quadratic	0.133	0.042	1,824.0	3.17	.002
Rhyme: Quadratic	0.104	0.042	1,824.0	2.48	.013
RD‐only: Cohort: Intercept	−0.028	0.026	304.0	−1.07	.283
RD + DLD: Cohort: Intercept	0.029	0.030	304.0	0.96	.336
RD‐only: Rhyme: Intercept	0.004	0.026	304.0	0.17	.863
RD + DLD: Rhyme: Intercept	0.027	0.030	304.0	0.92	.360
RD‐only: Cohort: Linear	−0.160	0.052	304.0	−3.07	.002
RD + DLD: Cohort: Linear	−0.108	0.061	304.0	−1.78	.076
RD‐only: Rhyme: Linear	0.050	0.052	304.0	0.96	.337
RD + DLD: Rhyme: Linear	0.088	0.061	304.0	1.45	.148
RD‐only: Cohort: Quadratic	−0.028	0.047	1,824.0	−0.59	.553
RD + DLD: Cohort: Quadratic	−0.125	0.055	1,824.0	−2.25	.025
RD‐only: Rhyme: Quadratic	−0.099	0.047	1,824.0	−2.09	.037
RD + DLD: Rhyme: Quadratic	−0.110	0.055	1,824.0	−1.99	.047

Note

Parameters for the RD‐only and RD + DLD groups are estimated relative to the TD group, whereas parameters for the cohort and rhyme item pair conditions are estimated relative to the unrelated item pair condition.

To address our study hypotheses, we first examined learning trajectories for cohort and rhyme item pairs relative to unrelated item pairs, and evaluated whether these trajectories differed between the RD‐only and TD groups. We predicted that relative to the TD group, the RD‐only group would show: (i) reduced growth for cohort item pairs compared to unrelated item pairs, and (ii) enhanced (or equivalent) growth for rhyme item pairs relative to unrelated item pairs. Our observations were in line with these predictions. First, as reported in Table 2, for the RD‐only group, the slope of learning for cohort item pairs relative to unrelated item pairs was significantly shallower compared to the TD group (*Estimate* = −0.160; *SE* = 0.052; *p* = .002). Second, the quadratic term for rhyme item pairs relative to unrelated item pairs was significantly more negative for the RD‐only group compared to the TD group (*Estimate* = −0.099; *SE* = 0.047; *p* = .037), indicating a sharper rise and fall, or greater curvature, in the trajectory of learning. As illustrated in Figure 2, this greater curvature indexes a relative lack of difference between rhyme and unrelated item pairs in the RD‐only group compared to the TD group, reflective of an enhanced growth trajectory for rhyme item pairs in the RD‐only group.

Next, we examined learning trajectories for all three stimulus types, and evaluated whether these trajectories differed between the RD + DLD and TD groups, and between the RD + DLD and RD‐only groups. We predicted that (i) relative to the TD group, the RD + DLD group would show analogous differences in learning trajectories for cohort and rhyme item pairs as the RD‐only group, and (ii) relative to both the TD group and the RD‐only group, the RD + DLD group would show reduced growth for unrelated item pairs. Our observations were in line with these predictions. First, we found that for the comorbid RD + DLD group, the quadratic term for rhyme item pairs relative to unrelated item pairs was significantly more negative compared to the TD group (*Estimate* = −0.110; *SE* = 0.055; *p* = .047), indicative of an enhanced growth trajectory for rhyme items pairs. However, for cohort item pairs, the quadratic term was significantly more negative than that of the TD group (*Estimate* = −0.125; *SE* = 0.055; *p* = .025), indicating a sharper peak. Furthermore, when the RD‐only and RD + DLD groups were directly compared using a similar model containing only these two groups, the RD + DLD group had a significantly higher intercept for cohort item pairs (*Estimate* = 0.056; *SE* = 0.023; *p* = .013), as well as a significantly more negative quadratic term for cohort item pairs (*Estimate* = −0.097; *SE* = 0.043; *p* = .024). This pattern is indicative of enhanced growth for cohort item pairs compared to the RD‐only group, which was unexpected. Yet, rather than this solely reflecting a difference in relative difficulty for learning cohort item pairs, it is possible this pattern instead reflects a difference between groups in the learning trajectories for unrelated item pairs. Indeed, when similar models were performed using only the unrelated condition, the RD + DLD group showed a significantly lower intercept (*Estimate* = −0.099; *SE* = 0.031; *p* = .002), shallower slope (*Estimate* = −0.115; *SE* = 0.048; *p* = .018), and more positive quadratic term (*Estimate* = 0.104; *SE* = 0.038; *p* = .006) relative to the TD group, indicative of reduced growth. In contrast, the RD‐only group only showed a marginally more positive quadratic term relative to the TD group (*Estimate* = 0.058; *SE* = 0.032; *p* = .071). Furthermore, when the RD‐only group was directly compared to the RD + DLD group, the RD + DLD group showed a significantly lower intercept (*Estimate* = −0.074; *SE* = 0.024; *p* = .002) and marginally shallower slope (*Estimate* = −0.067; *SE* = 0.037; *p* = .071) for unrelated item pairs.

#### 3.1.1 Exploratory analysis of the role of ADHD symptomatology

Although not of primary interest in this study, given the (typical) high degree of comorbid ADHD in this sample and the hypothesized potential that inattention could impact learning trajectories, we also conducted an exploratory analysis to evaluate whether ADHD symptomatology may have contributed to any of the observed effects. In this analysis, we re‐ran the model with just the RD‐only and RD + DLD learner groups and included ADHD status, as well as its interaction with learner group, stimulus type, or both learner group and stimulus type, as additional fixed effects. In this model, all interactions including ADHD status, as well as the main effect of ADHD status, were not significant (all *p* > .15).

#### 3.1.2 Reading skill and age‐matched subgroup analysis

**Figure 3:**
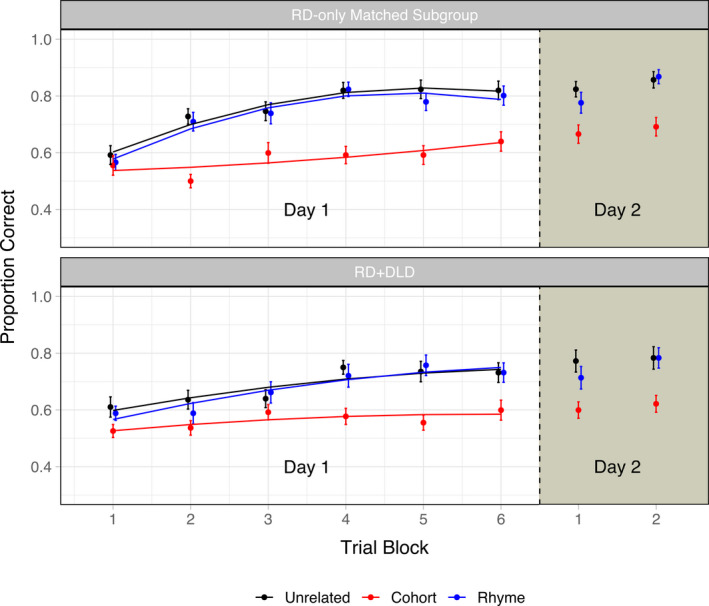
Accuracy across blocks for the artificial lexicon learning experiment for the RD + DLD group alongside the subgroup of 34 children from the RD‐only group who were reading skill and age‐matched to the RD + DLD group. The first six blocks constitute the first day of learning, whereas the last two blocks (i.e. to the right of the dashed line) constitute the second day. Points and standard error bars represent raw data, whereas solid lines represent model fits

A potential concern regarding these analyses is that in addition to comorbid language impairments, children in the RD + DLD group differed from the RD‐only group in that they had lower mean reading scores as well as a higher mean age. Therefore, it is difficult to tease apart the effects of comorbidity of RD and DLD from the effects of severity of reading deficits. For this reason, we selected a subset of 34 children (using version 3.0.2 of the R package *MatchIt*; Ho, Imai, King, & Stuart, 2011) from the RD‐only group who were matched to the RD + DLD group in age and reading skills as well as the number of children with an ADHD diagnosis. We then performed similar models as those reported above to compare the RD + DLD group to the subset of children from the RD‐only group (Figure 3). Results were similar to the previous set of analyses: When the RD‐only and RD + DLD groups were compared directly, the RD + DLD group showed a significantly higher intercept for cohort item pairs relative to unrelated item pairs (*Estimate* = 0.056; *SE* = 0.025; *p* = .029), and when learning trajectories for just the unrelated item pairs were compared between the two groups, the RD + DLD group showed a lower intercept compared to the RD‐only group (*Estimate* = −0.071; *SE* = 0.029; *p* = .015). Additionally, when the exploratory analysis including ADHD status was repeated for these subgroups, no main effects or interactions including ADHD status were significant (all *p* > .07).^3^

### 3.2 Retention of items on Day 2

**Figure 4:**
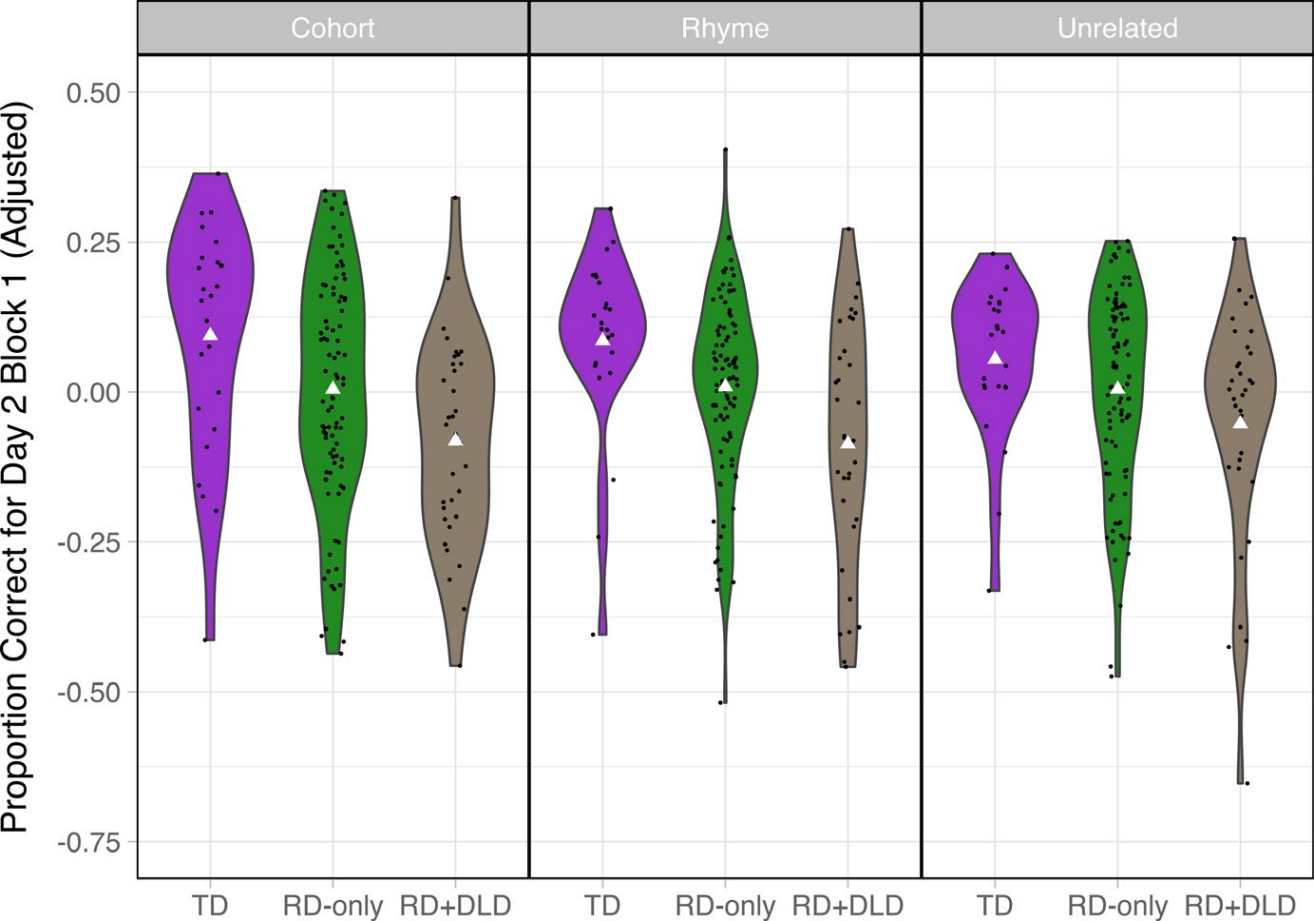
Adjusted Day 2 Block 1 accuracy (residualized on Day 1 learning estimates as well as the amount of time elapsed between Day 1 and Day 2 testing sessions) for the three learner groups and three stimulus types. Note that because accuracy values are residualized, they can be negative. Individual data points are jittered within the violin plots. The white triangles indicate group means for each stimulus type

To address our hypotheses concerning the effects of offline consolidation, we evaluated whether retention differed across learner groups for all three stimulus types, as measured by Day 2 Block 1 discrimination accuracy residualized on Day 1 learning estimates for each stimulus type and each learner group as well as the amount of time elapsed between Day 1 and Day 2 testing sessions (Figure 4). We predicted that relative to TD children, children with RD would show poorer retention of cohort and rhyme item pairs but not unrelated item pairs, whereas children with comorbid RD and DLD would show poorer retention for all three stimulus types. Our observations were mostly in line with these predictions. First, multiple regression analysis revealed that retention of cohort item pairs was significantly lower in the RD‐only group compared to the TD group (*Estimate* = −0.089; *SE* = 0.043; *p* = .038), and in the RD + DLD group relative to the TD group (*Estimate* = −0.176; *SE* = 0.050; *p* < .001), even when including parameter estimates characterizing Day 1 learning as predictors in the model. Similarly, retention of rhyme item pairs was significantly lower in the RD‐only group compared to the TD group (*Estimate* = −0.076; *SE* = 0.038; *p* = .047), and significantly lower in the RD + DLD group relative to the TD group (*Estimate* = −0.172; *SE* = 0.044; *p* < .001). For unrelated item pairs, only the RD + DLD group showed significant differences in retention compared to the TD group (*Estimate* = −0.108; *SE* = 0.044; *p* = .016). Second, when the RD‐only and RD + DLD groups were directly compared to each other using a similar modeling approach (i.e. using parameter estimates for learning growth curves from a model containing only these two groups), the RD + DLD group showed significantly lower retention of cohort and rhyme item pairs compared to the RD‐only group (cohort item pairs: *Estimate* = −0.082; *SE* = 0.038; *p* = .032; rhyme item pairs: *Estimate* = −0.091; *SE* = 0.034; *p* = .009), as well as marginally lower retention of unrelated item pairs (*Estimate* = −0.059; *SE* = 0.035; *p* = .098).

#### 3.2.1 Reading skill and age‐matched subgroup analysis

**Figure 5:**
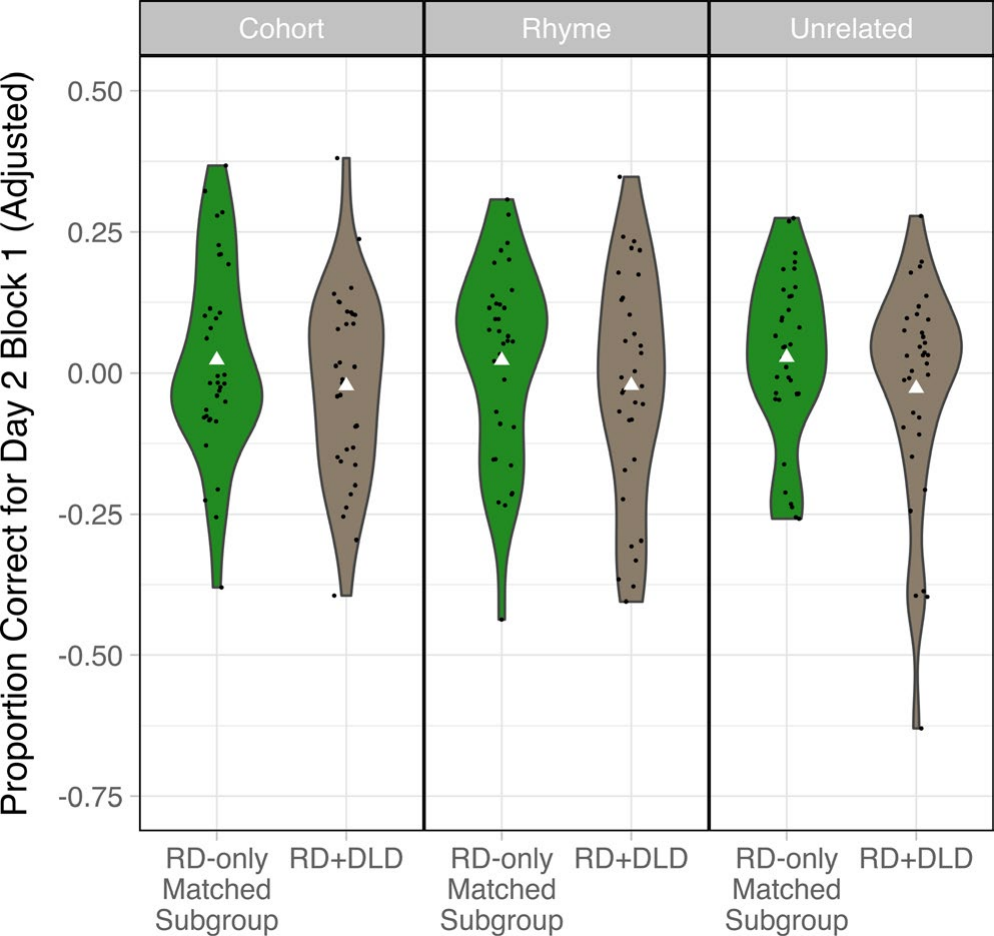
Adjusted Day 2 Block 1 accuracy (residualized on Day 1 learning estimates as well as the amount of time elapsed between Day 1 and Day 2 testing sessions) for the RD + DLD group alongside the subgroup of 34 children from the RD‐only group who were reading skill and age‐matched to the RD + DLD group. Note that because accuracy values are residualized, they can be negative. Individual data points are jittered within the violin plots. The white triangles indicate group means for each stimulus type

When similar analyses were repeated with the subgroup of 34 children from the RD‐only group who were reading skill and age‐matched to the RD + DLD group (Figure 5), the RD + DLD group did not show significant differences in retention of cohort, rhyme, or unrelated item pairs compared to the RD‐only group (cohort item pairs: *Estimate* = −0.047; *SE* = 0.043; *p* = .271; rhyme item pairs: *Estimate* = −0.046; *SE* = 0.047; *p* = .331; unrelated item pairs: *Estimate* = −0.057; *SE* = 0.044; *p* = .198).

## 4 DISCUSSION

In this study, we assessed how RD and comorbid DLD impact how children learn and remember words with different phonological relationships. This was motivated by work documenting associations between word learning and reading and language skills (Alt et al., 2017, 2019; James et al., 2019; Kan & Windsor, 2010; Kimppa et al., 2018; Landi et al., 2018; Litt et al., 2019; Smith et al., 2018), as well as by studies suggesting that the stability of phonological representations may be compromised in RD and DLD (Desroches et al., 2006, 2013; Kornilov et al., 2015; Malins et al., 2013; McMurray, Klein‐Packard, & Tomblin, 2019; McMurray et al., 2010). To address this aim, we measured learning as well as offline consolidation of an artificial lexicon of spoken pseudowords. This artificial lexicon consisted of a closed set of phonologically similar pseudowords including pairs of items that shared either word onset (cohort item pairs), word‐final information (rhyme item pairs), or were phonologically unrelated. We hypothesized that: (i) compared to TD children, children with RD would show reduced learning of cohort item pairs, suggesting increased cohort interference, and typical or enhanced rhyme learning, suggesting reduced rhyme interference; (ii) children with comorbid RD and DLD would show broader impairments in word learning compared to children with only RD, which would manifest as reduced learning for unrelated item pairs in addition to cohort and rhyme item pairs; (iii) analogous patterns of differences across learner groups also would be apparent in offline consolidation even when accounting for potential differences in Day 1 learning.

### 4.1 Differences in word learning across learner groups

As hypothesized, we observed differences in the trajectory of word learning across learner groups. First, compared to TD children, children with RD showed a significantly reduced learning trajectory for cohort item pairs relative to unrelated item pairs. The reduced growth for cohort item pairs could reflect greater confusability for items that overlap in onset. This finding is consistent with Desroches et al. (2013), who observed that children with RD showed stronger cohort mismatch effects during a later time window of the N400 component during a picture‐spoken word matching ERP task. This effect was thought to indicate a lack of top‐down activation of cohort competitors prior to spoken word presentation, resulting in a greater reliance on bottom‐up information and consequently greater difficulty in resolving competition between words sharing onset.

Second, compared to TD children, children with RD showed a learning trajectory for rhyme item pairs that was more similar to that of unrelated item pairs than it was in the TD group, suggesting that rhyme interference had a smaller impact in children with RD. Differences in rhyme interference have been observed previously during spoken word processing in children using eyetracking (Desroches et al., 2006) and ERPs (Desroches et al., 2013). Similar patterns also have been observed in a community sample of young adults using eyetracking measures collected during the same artificial lexicon learning paradigm that was employed in the current study (Magnuson et al., 2011). Desroches et al. (2006) attributed their results to a lack of sensitivity to rhyme relationships during spoken word recognition in children with RD, stemming from a lack of top‐down activation of word‐final phonemes prior to receiving spoken word input (Desroches et al., 2013). This weaker top‐down modulation during phonological processing also is supported by differences in brain connectivity between TD children and children with RD during a rhyme judgment task (Cao, Bitan, Chou, Burman, & Booth, 2006).

However, differences in top‐down processing are not the only possible explanation to account for this pattern of effects. For example, using simulations of the TRACE model of speech perception (McClelland & Elman, 1986), Magnuson et al. (2011) reported that reduced rhyme competition effects also can result from reduced lateral inhibition amongst phonemic representations. Yet, as Magnuson et al. (2011) point out, this same manipulation did not result in differences in simulated cohort effects. Therefore, although this explanation of reduced lateral inhibition among phonemic representations is consistent with Magnuson et al. (2011), who did not observe any evidence of cohort effects in their data, it is inconsistent with the current results, in which we did observe a significantly reduced learning trajectory for cohort item pairs relative to unrelated item pairs in the group of children with RD compared to the group of TD children. For this reason, we assert that the best explanation for the observed learning differences between the group of children with RD and the group of TD children is that the group of children with RD exhibited a lack of top‐down activation of phonemic information (in word‐initial and word‐final positions) prior to spoken word presentation, which translated to a greater dependence on bottom‐up information once target words unfolded (Desroches et al., 2013).

In addition to the noted differences in learning trajectories for cohort item pairs, the current findings are also somewhat inconsistent with the general learning effects observed in Magnuson et al. (2011). In that report, the authors observed overall differences in slopes between learner groups when collapsing across stimulus types. A potential explanation for this difference across studies could be differences in the type and origin of reading impairments: although the community sample in Magnuson et al. (2011) tended to have lower than average reading scores, the etiology of their reading difficulties is unknown. Furthermore, Magnuson et al. (2011) tested adults, so observed differences between our findings and theirs may be developmental or due to differences in reading experience.

Next, turning to the group of children with comorbid RD and DLD, we observed that similar to the group of children with only RD, learning trajectories for rhyme and unrelated item pairs were indistinguishable from each other. This finding of a lack of rhyme interference in the group of children with RD and DLD complements earlier results from Malins et al. (2013), who observed no attenuation of the N400 for rhyming compared to unrelated words in children with DLD compared to TD children. Similar to what has been proposed in RD, these differences in rhyme effects may be the result of reduced top‐down processing in children with DLD compared to TD children (Weismer, Plante, Jones, & Tomblin, 2005). However, it is also possible that the observed differences in rhyme processing may instead be the result of broader impairments in those with comorbid RD and DLD (Alt et al., 2019; Bishop & Snowling, 2004; Catts et al., 2005; Ramus et al., 2013). This is supported by the finding that the intercept for unrelated item pairs was lower in the group of children with comorbid RD and DLD relative to the group of children with only RD, yet was not different between the group of children with only RD and the group of TD children. This was observed in both the full sample analysis as well as in the reading and age‐matched subgroup analyses, suggesting that the presence of concurrent oral language impairments is a more likely explanation for this effect as opposed to more severe reading deficits in the group of children with comorbid RD and DLD. This reduction in performance for unrelated item pairs suggests that word learning difficulties in children with RD and DLD may extend beyond items sharing phonological similarity. In turn, because the unrelated condition was treated as the baseline condition to which the cohort and rhyme conditions were compared, a reduction in the learning rate for unrelated item pairs could account for the smaller difference in learning rates between the cohort and unrelated conditions that was observed in the group of children with comorbid RD and DLD compared to the other two learning groups.

This view of broader impairments underlying comorbid RD and DLD is supported by Alt et al. (2019), who observed that children with both RD and DLD showed deficits in phonological and semantic tasks compared to TD children, whereas children with RD only showed deficits in phonological tasks. This view is also partially supported by McMurray et al. (2010), who observed that compared to TD individuals, adolescents with DLD showed increased competition between targets and cohort or rhyme competitors during later stages of the time course of spoken word processing. The authors attributed this finding to faster decay of lexical information in short‐term memory in individuals with DLD, a view that also has been supported by MEG results (Helenius, Parviainen, Paetau, & Salmelin, 2009). More recently however, McMurray et al. (2019) assert that spoken word processing deficits in DLD may instead result from a lack of inhibition between word‐level representations in DLD. As McMurray et al. (2019) acknowledge, these explanations are not necessarily mutually exclusive. Moreover, because we only used this specific task to examine learning, we cannot rule out the possibility that the observed pattern of results could reflect domain‐general learning deficits in DLD that extend beyond phonological‐lexical processing (Ullman, Earle, Walenski, & Janacsek, 2020).

Although we cannot adjudicate amongst these possible explanations based on the data we collected, the current study nonetheless adds to a growing literature documenting word learning deficits in children with RD and/or DLD compared to TD children (Alt et al., 2017, 2019; James et al., 2019; Kan & Windsor, 2010; Kimppa et al., 2018; Landi et al., 2018; Litt et al., 2019). Furthermore, the current findings help clarify the contexts in which potential word learning deficits are apparent. First, although it has been claimed that paired associate learning deficits in RD are restricted to tasks that involve output phonology (Litt et al., 2019), we observed word learning deficits in a task involving verbal‐visual paired associated learning. We suggest that the artificial lexicon learning task used in this study provides increased sensitivity to detect differences in input phonology during paired associate learning across learner groups relative to other PAL tasks that have been used in the past.

It is also worth noting that we did not uncover strong evidence that concurrent attention deficits were associated with word learning in the current study. Although exploration of ADHD was not a primary aim of the study, it is important to consider given the high comorbidity between RD and ADHD (Willcutt & Pennington, 2000). Furthermore, ADHD has previously been associated with deficits in implicit learning (Barnes, Howard, Howard, Kenealy, & Vaidya, 2010). However, based on the current results, we assert that it is unlikely that concurrent attention deficits can account for all the differences in phonological‐lexical processing that we observed in children with reading and language impairments.

### 4.2 Differences in offline consolidation across learner groups

In addition to differences in word learning, we also observed deficits in offline consolidation between learner groups in the full sample analysis. Specifically, compared to the TD group, children with RD and comorbid RD and DLD showed lower retention of cohort and rhyme item pairs. Furthermore, when the group of children with comorbid RD and DLD were compared to the group of children with only RD, the children with comorbid RD and DLD showed even lower retention of cohort and rhyme item pairs. However, when we ran reading and age‐matched subgroup analyses, these differences between subgroups were not as apparent as they were with the full sample, suggesting that some of the groupwise differences that were observed in the full sample may have been driven by the severity of reading deficits rather than comorbidity between reading and language impairments. Previous research suggests that offline consolidation may be important for establishing facilitatory and inhibitory connections among phonologically similar words, which may lead to increased lexical competition effects (Davis & Gaskell, 2009; Dumay & Gaskell, 2007). Although our findings are not fully consistent with enhancements for phonological (or phonological to semantic) connections following a period of offline consolidation, the groupwise differences in retention for the full sample could have arisen due to differences in maintenance of newly established connections among ‘lexical’ constituents. These findings extend upon previous observations of differences in consolidation effects for RD and DLD (Earle et al., 2018; McGregor et al., 2013; Smith et al., 2018) and links between individual differences in consolidation and reading skill among TD adults (Landi et al., 2018).

### 4.3 Limitations and implications

The current set of findings suggests that the presence of concurrent oral language and reading deficits contributes to word learning difficulties beyond the presence of a reading deficit alone. However, because the current study did not include a group of children with only DLD, we are not in a position to make definitive claims regarding whether it is specifically the presence of oral language impairments that resulted in the difficulties observed in the group of children with comorbid RD and DLD, or whether the difficulties observed in this group can instead be attributed to impairments underlying comorbid cases that may be different in origin from the impairments associated with RD or DLD alone (Pennington & Bishop, 2009).

In addition to limitations with respect to the study sample, there are also some limitations with respect to the measures acquired. More specifically, we collected accuracy at the trial level, but did not collect online measures such as eyetracking and ERPs that have previously been used to study the temporal dynamics of phonological competition (Desroches et al., 2006, 2013; Kornilov et al., 2015; Magnuson et al., 2011; Malins et al., 2013; McMurray et al., 2010). Nevertheless, the observed pattern of results was quite consistent with these previous studies, suggesting the current paradigm has the potential to reveal meaningful differences between groups even in the absence of online measures. This may be especially valuable in settings in which the collection of online measures is not feasible. With that said, our view is that future studies should build on the current results by using online measures to characterize the nature of within‐trial effects during word learning.

Several aspects of the present design also limit strong conclusions regarding overnight consolidation. First, because Day 1 and Day 2 learning occurred during the school day between 8 a.m. and 5 p.m., this allowed for individual differences in the time delay and the amount of intervening linguistic information between testing sessions, both of which may have mediated offline consolidation effects (Earle & Myers, 2013; Walker et al., 2020). For this reason, we included the amount of time elapsed between testing sessions as an additional predictor of non‐interest when analysing retention data. A second limitation is that we did not measure sleep duration or sleep quality, which also have been shown to influence consolidation effects (Earle, Landi, & Myers, 2017; Earle & Myers, 2014; Smith et al., 2018). Finally, without online processing measures, it is difficult to tease apart whether decreased performance on the second day of testing reflected poorer retention or was instead the result of increased phonological competition between items following lexicalization (e.g. Weighall, Henderson, Barr, Cairney, & Gaskell, 2017).

Despite these limitations, there are several potential implications from the current set of findings. First, from an educational perspective, the current findings can contribute to models of vocabulary acquisition that take into account child‐level and word‐level factors in order to identify optimal instructional strategies for individual learners (Elleman, Steacy, Olinghouse, & Compton, 2017). These models may highlight the need for additional exposures and learning trials for those students with both reading and language impairments, and may motivate careful selection of the specific vocabulary focus for individual learners in light of potential competition effects. In addition, the current methodology of tracking spoken artificial lexicon learning over time may provide an approach for dynamic assessment over the course of word learning, especially with respect to onset and rime discrimination skills that may play important (and potentially differential) roles in terms of remediation for struggling readers (Lovett, Lacarenza, & Borden, 2000). Second, from a clinical perspective, differences in how phonological similarity impacts word learning may help illuminate the ways in which concurrent reading and language impairments impact vocabulary acquisition compared to vocabulary learning with just reading impairments alone. These individual differences across learners could be particularly important when considering reading and vocabulary development in children, as the effects of phonological similarity on lexical competition become more pronounced as neighbourhoods become denser (Walley, Metsala, & Garlock, 2003), and early differences in spoken vocabulary growth will not only impact the acquisition of literacy (Anthony & Francis, 2005) but also compound over the course of reading development (Cain & Oakhill, 2011).

## 5 CONCLUSIONS

Using an artificial lexicon learning paradigm with an overnight design, we acquired evidence that: (i) children with RD show difficulties compared to TD children in learning and remembering phonologically similar pseudowords; (ii) children with comorbid RD and DLD show broader deficits in both online word learning and offline consolidation compared to children with only RD. Although word learning difficulties have been previously associated with RD (Alt et al., 2017, 2019; Kimppa et al., 2018; Smith et al., 2018), the current study offers evidence that the underlying impairments associated with RD impact both online learning and offline consolidation of newly learned words, a finding that can inform extant theories and models of RD. Furthermore, the current findings contribute to our understanding of the specific learning deficits underlying comorbid RD and DLD by suggesting that children with concurrent oral language and reading deficits may have broader impairments that impact their ability to establish robust phonological‐lexical representations (Alt et al., 2019). To build on these findings, it is our view that future research should clarify the cognitive and neurobiological foundations of word learning in order to better understand how children with either or both of these learning disabilities make use of phonological similarity to guide the organization of new word knowledge.

## CONFLICT OF INTEREST

The authors declare no competing financial interests.

## Data Availability

Preprocessed data and analysis scripts are available on the following Open Science Framework project page: https://osf.io/az3tf/.

## ENDNOTES

^1^ Note that reading disability may be called dyslexia or specific reading disability (SRD) in other research, and that developmental language disorder may be called specific language impairment (SLI) or language impairment (LI). We are using RD and DLD throughout for the sake of consistency, even when talking about the results from other studies that may not have used these labels. It should also be noted that unlike some other studies, we are not using definitions for RD or DLD that are based on discrepancies between reading or oral language skills and IQ measures. For this reason, we did not exclude participants on the basis of IQ measures. However, we did repeat all analyses excluding data from two participants who had Full Scale IQ measures from the Wechsler Abbreviated Scale of Intelligence (WASI‐II; Wechsler, 2011) that were less than 80 (although both participants had at least one subtest with a T‐score of 40 or above, corresponding to low average scores). When we excluded these participants, we observed similar results for all analyses.

^2^ Based on previous literature suggesting that strong competition is not predicted on the basis of phonemic overlap in medial positions of spoken words (Allopenna et al., 1998; Magnuson et al., 2003), we did not predict differences in learning trajectories for fully unrelated item pairs compared to near neighbour item pairs. In addition, when we ran a growth curve analysis comparing learning trajectories between these two stimulus types, there were no significant differences between the three learner groups (all *p*> .05). For these reasons, we elected to combine trials for these two item types when computing accuracy across blocks for unrelated item pairs. This resulted in the number of trials for unrelated item pairs being equivalent to the number of trials for cohort and rhyme pairs, thus allowing for the unrelated item pair condition to serve as a stable baseline condition in the growth curve analyses that we subsequently conducted.

^3^ This model initially gave rise to a singular fit. As a result, random slopes for stimulus types nested within participants were removed from the model.
